# Assessment of Serum Cytokines and Oxidative Stress Markers in Elite Athletes Reveals Unique Profiles Associated With Different Sport Disciplines

**DOI:** 10.3389/fphys.2020.600888

**Published:** 2020-10-15

**Authors:** Muhammad U. Sohail, Layla Al-Mansoori, Hend Al-Jaber, Costas Georgakopoulos, Francesco Donati, Francesco Botrè, Maha Sellami, Mohamed A. Elrayess

**Affiliations:** ^1^Biomedical Research Center, Qatar University, Doha, Qatar; ^2^Anti-Doping Laboratory Qatar, Doha, Qatar; ^3^Laboratorio Antidoping, Federazione Medico Sportiva Italiana, Rome, Italy; ^4^College of Art and Science, Sport Science Program, Qatar University, Doha, Qatar

**Keywords:** elite athletes, cytokines, oxidative stress, biomarkers, power, cardiovascular demand, endurance

## Abstract

**Objectives:**

Circulating cytokines and oxidative stress markers vary in response to different exercise regimens. This study aims to compare the immune-inflammatory and oxidative stress profiles of elite athletes from different sport disciplines as potential biomarkers of muscle damage, and cardiovascular demand.

**Methods:**

Serum samples from 88 consented elite male athletes from different sports disciplines (aquatics, *n* = 11, athletics, *n* = 22, cycling, *n* = 19, football, *n* = 28 and weightlifting, *n* = 8) collected at the anti-doping lab in Italy were screened for 38 cytokines and oxidative stress markers. Comparisons were made between different level of power, cardiovascular demand (CD) and endurance, as well as among the sport types.

**Results:**

The anti-inflammatory interleukin (IL)-10 was higher (*p* = 0.04) in moderate power compared with the high power group. Conversely, superoxide dismutase (SOD; *p* = 0.001) and malondialdehyde (MDA; *p* = 0.007) levels were greater in the higher power groups compared with the lower power counterpart. Among athletes who belong to different CD ranks, IL-1β and monocyte chemoattractant protein-1(MCP1) levels were higher (*p* = 0.03) in the low CD-rank group compared with high CD counterpart, whereas, SOD levels were higher (*p* = 0.001) in high and moderate CD-rank groups compared to low counterpart. For endurance groups, IL-10 and macrophage inflammatory protein (MIP)-1beta were increased (*p* = 0.03) in low/moderate endurance compared with the high endurance group. Finally, MIP1-beta, SOD and catalase varied significantly among the sports groups.

**Conclusion:**

Specific markers of inflammation and oxidative stress are associated with different sports disciplines and could be utilized as potential biomarkers of athletes’ health, performance, and recovery from injury.

## Introduction

Long term physical training of professional athletes results in a wide spectrum of structural and biochemical adaptations in body organs and systems. Specific musculoskeletal and cardiopulmonary responses are observed in athletes, which mostly depend upon the type and duration of their training (Schumann et al., 2015). These morphological adaptations are essentially triggered by metabolic, immune-inflammatory, and neuroendocrine modifications. In particular, strenuous training in endurance athletes significantly alters their metabolic profile characterized by enhanced steroid biosynthesis, fatty acid metabolism, oxidative stress, and energy homeostasis (Al-Khelaifi et al., 2018, 2019). These metabolic changes are strongly associated with physical training pattern and cardiovascular demand, therefore, offer an opportunity to assess athlete’s performance and their cardiovascular and musculoskeletal transformations (Al-Khelaifi et al., 2019). Recently, immune-inflammatory and oxidative stress markers are used to assess cardiopulmonary transformation, physical performance, and recovery during training (Lee et al., 2017).

Circulating cytokines and chemokines are reported to be good markers of cardiovascular demand (CD) and cardiorespiratory fitness (Jürimäe et al., 2018). However, the immune-inflammatory responses, as elicited by cytokine profiling, may differ in different physical training type. The practice of physical activity, regularly and without excess, stimulates the immune system. According to several reports, it has been shown that the practice of an intensive sport activity has the opposite effect of suppressing the immune system (Walsh et al., 2011). Unlike a sedentary lifestyle, regular moderate exercise reduces the risk of infection, but very prolonged exercise sessions and periods of intensive training or competition are associated with an increased risk of infection. In athletes, symptoms of respiratory disease are routinely observed as they approach competition, which can affect their exercise performance (Bermon, 2007; He et al., 2013).

Jürimäe et al. (2018) observed that exercise performance is associated with enhanced secretions of interleukin (IL)-2, IL-6, IL-8, vascular endothelial growth factor (VEGF), and monocyte chemoattractant protein-1 (MCP-1) (Jürimäe et al., 2018). These and many other immune-inflammatory cytokines can trigger a broad range of actions that require further elucidation in different sport training formats. For example, enhanced immune-inflammatory cascade during intensity training promotes IL-6 secretions and prevents tumor necrosis factor- (TNF) α and IL-1 production, which eventually support skeletal muscle growth and injury repair (Pedersen, 2000). Rong et al. (2008) suggested that secretion of cytokines in strength (fencing and kickboxing), static (shooting teams), and ball (football and volleyball) sports is regulated by muscle damage, CD and pulmonary vital capacity during exercise (Rong et al., 2008).

Studies focusing on the immune-inflammatory profiles of athletes from different training formats have reported inconsistent findings with regard to cytokine expression and cardiovascular adaptations (Rong et al., 2008; La Gerche et al., 2015; Schumann et al., 2015; Kaya, 2016; Jürimäe et al., 2018). Furthermore, a holistic analysis of progressive and adaptive immune responses to meet the physical needs of the cardiopulmonary and musculoskeletal systems in elite athletes remains scarce (Campbell and Turner, 2018). This study aims to compare the immune-inflammatory and oxidative stress profiles of elite athletes participating in different sporting events and to draw an association between systemic cytokine levels and oxidative stress markers and their endurance/power and CD. Our hypothesis is that certain markers of inflammation are specifically associated with different sports disciplines and as such can be used as potential biomarkers of athletes’ health, performance, and recovery from injury.

## Materials and Methods

### Study Design

The present study included 88 consented elite male athletes from different sport disciplines [aquatics, *n* = 11, athletics, *n* = 22 (10 long distance 3,000 m+, 6 athletics with undetermined discipline, 2 marathon runners, 1 Jumps, 1 short, 1 middle and 1 long distance runner), cycling, *n* = 19, football, *n* = 28 and weightlifting, *n* = 8], who participated in national or international sports events and tested negative for doping use at the Anti-doping laboratory in Italy. Blood samples were collected from the athletes and immediately shipped on ice to the Anti-doping laboratories with anonymous labels. Once received, the samples were centrifuged to separate the serum. The only information available to the researchers were the type of the sport and participant’s sex due to the strict anonymization process adopted at Anti-doping laboratories and that dictated by study’s ethics. The study was conducted under the guidelines of the World Medical Association Declaration of Helsinki. All protocols were approved by the Institutional Research Board of Qatar University (QU-IRB 1277-E/20). Sport types were dichotomized into low, moderate and high dynamic or static groups based on dynamic maximum oxygen uptake (VO_2_) and static maximum voluntary contraction (MVC) components associated with their sports (Mitchell et al., 1985; Table 1). Sport types were also dichotomized into three cardiovascular demand ranks (low, moderate and high) as shown previously (Mitchell et al., 2005). In this study, few athletes belonged to low levels of endurance, therefore they were merged with the corresponding moderate class (Table 1).

**TABLE 1:** Classification of study participants into endurance groups (low/moderate and high endurance), power groups (low, moderate and high) and cardiovascular demand groups (low, moderate, and high, colored in white, light gray and dark gray shades, respectively) (Mitchell et al., 2005).

	**Low/Moderate endurance (*n* = 17) (<70% VO_2_)**	**High endurance (*n* = 71) (>70% VO_2_)**
High power (*n* = 40) (>50% MVC)	Weightlifting (*n* = 8)	Cycling (*n* = 19), Long distance athletics (*n* = 13)
Moderate power (*n* = 20) (20–50% MVC)	Athletics (*n* = 9)	Aquatics (*n* = 11)
Low Power (*n* = 28) (<20% MVC)		Football (*n* = 28)

*Nine athletics (six undetermined, one jump, one short distance and one middle distance) were placed in the moderate power/moderate endurance group, whereas the rest were clustered in the high endurance/high power group, including 10 long distance runners, two marathon runners and one long distance runner.*

### Assessment of Cytokine

Levels of 35 secreted cytokines, chemokines, and growth factors in serum samples were measured using Cytokine 35-Plex Human Panel (LHC6005M, Thermo Fisher Scientific) on Luminex^TM^ 200 platform (Luminex Corp., Austin, TX, United States) according to manufacturer’s instructions (Qin et al., 2019). Luminex xMAP^®^ bead-based 35-plex immunoassay simultaneously measured 35 analytes using xPONENT 4.2 software provided with the Luminex platform. The Human Cytokine Magnetic 35-Plex Panel is validated for the quantitative determination of serum epidermal growth factor (EGF), Eotaxin, fibroblast growth factor (FGF) basic, granulocyte colony-stimulating factor (G-CSF), granulocyte-macrophage colony-stimulating factor (GM-CSF), hepatocyte growth factor (HGF), Interferon (IFN)-α, IFN-γ, interleukin 1 receptor antagonist (IL-1ra), IL-1α, IL-1β, IL-2, IL-2r, IL-3, IL-4, IL-5, IL-6, IL-7, IL-8, IL-9, IL-10, IL-12 (p40/p70), IL-13, IL-15, IL-17A, IL-17F, IL-22, interferon gamma-induced protein 10 (IP-10), MCP-1, monokine induced by gamma interferon (MIG), macrophage inflammatory protein (MIP)-1α, MIP-1β, regulated on activation, normal T cell expressed and secreted (RANTES), TNF-α, and VEGF.

### Assessment of Oxidative Stress Markers

Enzyme linked immunosorbent Assay (ELISA) was performed to assess serum concentrations of superoxide dismutase (SOD), catalase (CAT), and malondialdehyde (MDA) according to manufacturers’ instructions. SOD activity was measured using colorimetric assay kit (EIASODC, Thermo Fisher Scientific, Waltham, MA, United States) in 96-well microplate read at 450 nm optical density (OD) (Call et al., 2017). Sandwich ELISA for CAT enzyme activity was performed using commercially available kit (ab171572, Abcam Inc., Cambridge, MA, United States) and the assay OD was measured at 450 nm (Dacrema et al., 2020). Competitive ELISA for MDA was performed using commercially available ELISA kit (ab238537, Abcam Inc., Cambridge, MA, United States) and the assay OD was measured at 450 nm (Jiao et al., 2020).

### Statistics Analysis

Analysis was carried out using IBM SPSS version 25 (NY, IBM Corp.), R version 3.6 (R Core Team) and SIMCA 16.0.1 software (Umetrics, Sweden). Comparisons were made among sport types, endurance, CD-Rank, and power levels as categorized in Table 1. Statistical differences were evaluated between the groups using non-parametric Kruskal–Wallis (KS) one-way analysis of variance a nd Mann–Whitney *U*-tests. Orthogonal partial least square discriminant analysis (OPLS-DA) was utilized using SIMCA software version 16.0.1 to examine the ability of measured analytes to discriminate study groups. Heatmaps were constructed for correlations between sports categories (CD-Rank, Endurance, and power) and study parameters using Spearman’s correlation analysis.

## Results

Out of the 35 investigated cytokines, only eighteen were accurately detected in addition to three oxidative stress markers (Supplementary Tables S1–S4), while the remaining cytokines were either absent or their concentrations were below the detection limits.

### Cytokines and Oxidative Stress Markers Differentiating Power Groups

**FIGURE 1:**
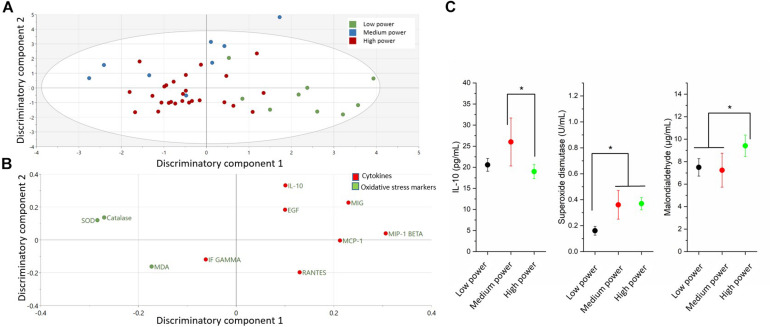
Differences in cytokines and oxidative stress markers among power groups (low, moderate and high). **(A)** The score plot of OPLS-DA model comparing analytes from three power groups exhibits class-discriminatory component 1 (*x*-axis) versus class-discriminatory component 2 (*y*-axis). **(B)** The corresponding loading plots shows top associated analytes differentiating low, moderate and high power groups. **(C)** Significant differences in cytokines and oxidative stress markers among the three power groups by KS test (*p < 0.05).

The comparison of cytokines and serum oxidative stress markers among the three power groups is presented in the Figure 1 and Table 1. An OPLS-DA was used to delineate a multivariate separation of low, moderate and high power elite athletes (Figure 1A). OPLS-DA exhibited two class-discriminatory components, accounting for 42% of the variation in the data due to participant groups. Principle component 1 on the *x*-axis divides low and high power groups, whereas principle component 2 on the *y*-axis separates the medium power group from the other two groups (Figure 1A). Top discriminatory analytes include IL-10, MIG, SOD, MIP-1β, and Catalase (Figure 1B). KS univariate analysis revealed elevated IL-10 levels in the moderate-power group compared with the high-power group (*p* = 0.04). Similarly, SOD activity was higher in the moderate- and high-power groups compared with the low-power group (*p* ≤ 0.001), whereas, MDA concentration was higher in the high compared to low- and moderate-power groups (*p* ≤ 0.008) (Figure 1C).

### Cytokines and Oxidative Stress Markers Differentiating Cardiovascular Demand Groups

**FIGURE 2:**
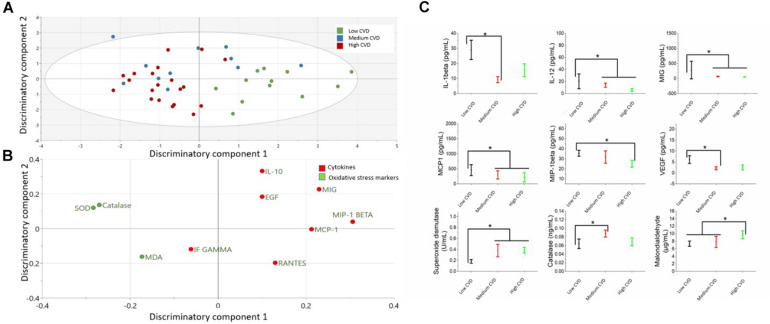
Differences in cytokines and oxidative stress markers among cardiovascular demand (CD) rank groups (low, moderate and high). **(A)** The score plot of OPLS-DA model comparing analytes from three power groups, exhibits class-discriminatory component 1 (*x*-axis) versus class-discriminatory component 2 (*y*-axis). **(B)** The corresponding loading plots showing top associated analytes differentiating low, moderate and high CD groups. **(C)** Significant differences in cytokines and oxidative stress markers among the three groups by KS test (*p < 0.05).

The comparison of cytokines and serum oxidative stress markers among CD-Rank groups is presented in the Figure 2 and Supplementary Table S2. OPLS-DA exhibited two class-discriminatory components, accounting for 40% of the variation in the data due to participant groups. Principle component 1 on the *x*-axis separates low CD-rank from medium and high groups (Figure 2A). Top discriminatory analytes include Catalase, SOD, MIG, MIP-1β, and IL-10 (Figure 2B). KS univariate analysis revealed elevated IL-1β (*p* = 0.04) and VEGF (*p* = 0.02) levels in the low CD-rank group compared with moderate group. Similarly, MIP-1β, IL-12, MIG, and MCP1 concentrations were higher in the low CD-rank group compared to moderate- and high-groups (*p* = 0.04), whereas, SOD (*p* ≤ 0.001) and CAT (*p* = 0.03) concentrations were high and moderate CD-rank groups compared to low group (Figure 2C).

### Cytokines and Oxidative Stress Markers Differentiating Endurance Groups

**FIGURE 3:**
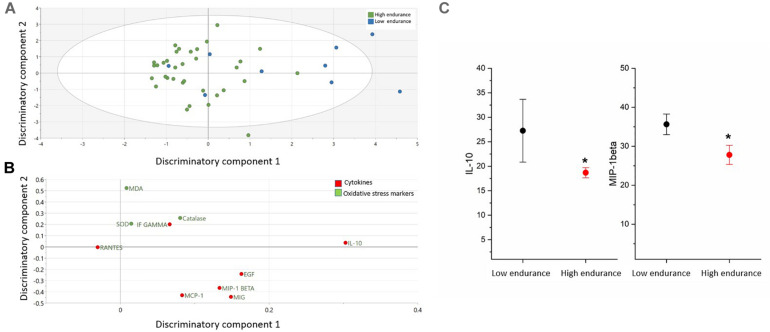
Differences in cytokines and oxidative stress markers between endurance groups (low and high). **(A)** The score plot of OPLS-DA model comparing analytes from two endurance groups exhibits one class-discriminatory component 1 (*x*-axis) versus an orthogonal component 2 (*y*-axis). **(B)** The corresponding loading plots shows top associated analytes differentiating low and high endurance groups **(C)**. Significant differences in cytokines and oxidative stress markers between the two groups by KS test (*p < 0.05).

The comparison of cytokines and serum oxidative markers among endurance is presented in the Figure 3 and Supplementary Table S3. OPLS-DA exhibited one class-discriminatory components, accounting for 35% of the variation in the data due to participant groups, whereas the experimental noise was captured in the form of an orthogonal component along the *y*-axis in Figure 3A. Principle component 1 on the *x*-axis separates low/moderate endurance groups from high endurance group (Figure 3A). Top discriminatory analytes include IL-10, MIG, EGF, MIP-1β, and MCP-1 (Figure 3B). KS univariate analysis revealed that only IL-10 and MIP-1β were significantly increased in low/moderate endurance compared with the high endurance group (*p* = 0.03) (Figure 3C).

### Cytokines and Oxidative Stress Markers Differentiating Athletes From Different Sport Groups

**FIGURE 4:**
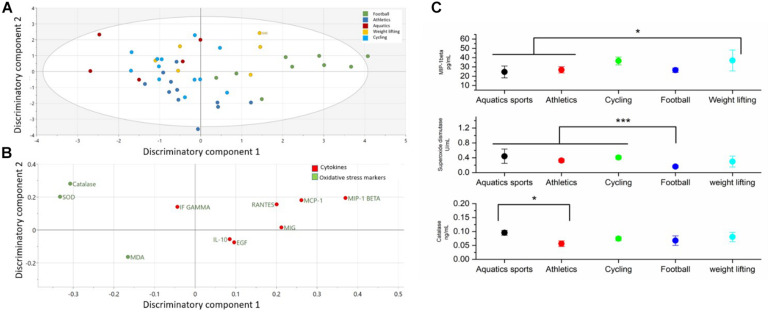
Differences in cytokines and oxidative stress markers among different sport groups. **(A)** The score plot of OPLS-DA model comparing analytes from five sport groups exhibits two class-discriminatory component 1 (*x*-axis) versus class-discriminatory component (*y*-axis) 2. **(B)** The corresponding loading plots shows top associated analytes differentiating five sport groups **(C)**. Significant differences in cytokines and oxidative stress markers between the two groups by KS test (*p < 0.05).

The comparison of cytokines and serum oxidative stress markers among sport groups is presented in the Figure 4 and Supplementary Table S4. OPLS-DA exhibited two class-discriminatory components, accounting for 26% of the variation in the data due to participant groups. Principle component 1 on the *x*-axis separates football from the rest, whereas principle component 2 on the *y*-axis separates weightlifting from the other sports (Figure 4A). Top discriminatory analytes include Catalase, MIP-1β, SOD, MCP-1, and RANTES (Figure 4). KS univariate analysis confirms that only MIP-1β, SOD, and catalase were significantly different among the sports groups. MIP-1β was significantly higher (*p* = 0.04) in the weightlifters and cyclists compared to the corresponding results of athletes performing aquatic sports and athletics. SOD on the other hand was significantly higher (*p* = 0.001) in the aquatic, athletics, and cycling groups compared to the football players. Aquatic sports exhibited higher catalase activity compared with the athletics group (*p* = 0.01) (Figure 4C).

### Correlation Analysis Between Sports Categories (CD-Rank, Endurance, and Power) and Detected Analytes

**FIGURE 5:**
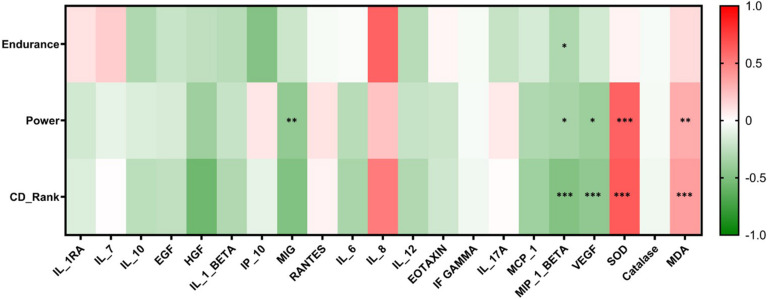
Heatmap reflecting the significant correlations between study groups and cytokines and oxidative stress markers. Correlations were made using spearman’s correlation analysis (*p ≤ 0.05, **p ≤ 00.01, ***p ≤ 00.001).

Correlation analysis was performed for CD-Ranks, power levels, and athlete’s endurance groups and the concentrations of the serum cytokines and oxidative stress markers. A negative correlation was observed between CD-Rank and both MIP-1β (*r* = −0.5, *p* ≤ 00.001) and VEGF (*r* = −0.4, *p* ≤ 00.001), whereas a positive correlation was seen between CD-Rank and both SOD (*r* = 0.6, *p* ≤ 00.001) and MDA (*r* = 0.4, *p* = 0.001). Similarly, a negative correlation was identified between increased power levels and serum concentrations of MIG (*r* = −0.4, *p* = 0.003), MIP-1β (*r* = −0.34, *p* = 0.02) and VEGF (*r* = −0.4, *p* = 0.02). Conversely, a positive correlation was identified between increased power levels and SOD (*r* = 0.6, *p* ≤ 00.001) and MDA (*r* = 0.3, *p* = 0.005). Furthermore, endurance levels were negatively correlated with MIP-1β (*r* = −0.3, *p* = 0.03). A heatmap summarizing associations between levels of power, endurance and CD rank and inflammatory and oxidative stress markers is shown below (Figure 5).

## Discussion

Excessive training in elite athletes, classified based on power, CD or endurance components, could aggravate their immune-inflammatory and oxidative stress responses. The knowledge of the pathophysiological adaptations to different types of exercise/sports is important for supervising training and managing injuries of elite athletes. Nevertheless, comprehensive scientific information concerning the immune-inflammatory and oxidative-stress responses during the process of adaptation in elite athletes are less described. Therefore, the present study compared cytokines and oxidative stress markers in the serum samples from elite male athletes collected at the Anti-doping laboratory in Italy. The athletes were dichotomized based on the static (%MVC) and dynamic (%VO_2_max) components of their respective sports into power, CD and endurance groups. Only 18 cytokines were accurately detected in addition to three oxidative stress markers. The emerging data revealed that markers of inflammation and oxidative stress varied among athletes from different sport groups and were uniquely associated with different sport disciplines. These unique signatures could reflect their training regiments, muscular injury and cardiovascular demands and could be utilized for managing their training protocols.

### Cytokines and Oxidative Stress Markers Differentiating Power Groups

In this study, serum anti-inflammatory cytokine IL-10 was higher in moderate power elite athletes, including athletics and aquatics, compared to medium power counterparts, including weightlifters, cyclists and long distance runners. Previous studies indicated that short exercise bouts induce the anti-inflammatory cytokine IL-10 (Steensberg et al., 2003; Lira et al., 2009), triggering an anti-inflammatory environment for several hours after the exercise (Windsor et al., 2018). High intensity interval exercise was also shown to induce IL-10 in lean and overweight–obese individuals (Dorneles et al., 2016). In contrast, other reports comparing elite athletes with untrained adults suggested no difference in IL-10 response to a single bout of exercise (Minuzzi et al., 2017). The emerging data also suggest that the higher power group is marked by increased oxidative stress marker MDA and increased activity of the anti-oxidative stress enzyme SOD compared to low power athletes. The heatmap confirms the significant positive correlations between increased power and SOD and MDA, but indicates a negative correlation with pro-inflammatory cytokines MIP-1β, MIG, and VEGF. MIP-1β and MIG represent inflammatory chemokines responsible for recruiting immune cells to damaged tissues, playing a role in the healing process (Wood et al., 2014; Hosaka et al., 2017). The elevated SOD and MDA levels in the high power group confirm previous reports indicating that acute aerobic exercise affects antioxidant levels and redox balance (Kawamura and Muraoka, 2018), which can be detected in the blood as it becomes a source of free radical production in erythrocytes and leukocytes (Nikolaidis and Jamurtas, 2009). Therefore, our emerging data may suggest that high power sports exhibit greater oxidative stress, less anti-inflammatory profile and lower tissue healing compared to moderate and low power sports.

### Cytokines and Oxidative Stress Markers Differentiating Cardiovascular Demand and Endurance Groups

Among the detected cytokines, the pro-inflammatory cytokines MIP1-β, IL-1β, IL-12, MIG, MCP1, and VEGF were higher in lower cardiovascular demand rank (rank 3), including footballers and athletes performing athletics, compared to higher cardiovascular demand ranks (ranks 4 and 5) that include weightlifters, aquatics, cyclists and long-distance runners. This was also evident in the heatmap as the MIP1-β, MCP1, and MIG were negatively correlated with increased cardiovascular demand but positively correlated with oxidative stress markers, such as SOD and MDA. MIP1-β is produced by macrophages following stress and stimulates oxidative stress system (Tatara et al., 2009; Casaletto et al., 2018). Similarly, the role of MIP1-β in regulating myoblast responses to skeletal muscle injury was previously described (Yahiaoui et al., 2008). IL-1β, released from blood monocytes, was previously shown to be elevated in muscle tissue in response to eccentric exercise (Cannon et al., 1989; Moldoveanu et al., 2000) and in the plasma of highly trained athletes during exercise together with IL-8 levels and histamine (Mucci et al., 2000). The pro-inflammatory cytokine IL-12 too was shown to increase immediately after a brief anaerobic maximal cycle ergometer exercise (Akimoto et al., 2000). MIG, a chemokine induced by IFN-γ, increases after two 90-min games separated by a 72 h in elite female football players (Andersson et al., 2010). The proinflammatory cytokine MCP-1 was also reported to increase in the endurance-exercise-trained athletes after exercise, with a suggested role in energy metabolism (Schild et al., 2016). VEGF produced by skeletal muscle cells and secreted into the circulation was also shown to be significantly increased following acute exercise in well-trained endurance athletes (Kraus et al., 2004). The elevated cytokines in cardiovascular demand groups 3 (footballers and athletics) over athletes with greater cardiovascular demand (weightlifters, aquatics, cyclists and long-distance runners) could reflect enhanced muscle injury associated with these groups. Interestingly, among the detected cytokines, only MIP1-β and IL-10 varied significantly between the endurance groups as they were elevated in moderate endurance (weightlifters and athletics) compared to high endurance group (cyclists, long distance runners, aquatics and footballers), also shown in the heatmap as MIP1-β correlated negatively with increasing endurance.

### Cytokines and Oxidative Stress Markers Differentiating Athletes Who Belong to Different Sport Groups

When comparing the serum levels of detected oxidative stress markers and cytokines in athletes who belong to five different sport groups (weightlifters, athletics including long distance runners, cyclists, aquatics and footballers), the anti-oxidative stress enzyme SOD was found to be decreased in footballers compared to aquatics, athletics and cycling. Previous studies have shown increased oxidative stress during exercise that affects performance by reducing the muscle contraction and causing fatigue (Powers and Jackson, 2008). The anti-oxidative enzyme CAT was too found to be increased in aquatics compared to athletics. Among detected cytokines, only MIP1-beta was increased in weightlifters compared to athletics and aquatics. Previously, Rong et al. (2008) observed changes in cytokines expression between different sports type. However, in comparison with the findings of our study, they observed higher level of IL-4 in swimmers and strength-sports players and a decrease in IL-10 compared with the control and the static-sports group. Similarly, many studies have also reported that the antioxidant enzyme activities (CAT and SOD) are higher in physically active people (swimmers and running athletes) compared with the controls (Nonato et al., 2016; Spanidis et al., 2017). However, more comprehensive comparisons are only evident when all sports are categorized according to endurance, power, and CD-Ranks (da Silva et al., 2016; Margaritelis et al., 2018).

### Study Limitations

Several confounders were unavoidable as blood samples were collected at different sites and multiple times (IN/OUT of competition, time of collection, latency time between training session and blood sampling). As a result, a potential batch effect might have hindered the associations between cytokines/oxidative stress markers and sport groups. Furthermore, there was no specific analysis differentiating athletes performing athletics that include various sporting events such running, jumping, throwing, and walking. This was mainly due to the limited availability of information about the athletes and their disciplines as mandated by the study ethics. Additionally, it should be noted that the analysis of the functional interrelationships between physical exercise and the immune system is extremely complex for two reasons: the multiplicity of factors intervening in this relationship (sleep, nutritional aspect, supplements/medication, type of exercise, frequency, intensity among others) and the variability of the individual parameters concerning the immune response which is established as an additional difficulty for the common validity of studies on this topic. Despite these confounders, clear cytokine and oxidative stress signatures were evident as the study was sufficiently powered, although further validation in larger cohorts at multiple times is warranted to confirm our findings.

### Study Strength and Novelty

This study highlights for the first time differences in cytokines and oxidative stress markers in elite athletes from different sports disciplines. Samples are often difficult to obtain from elite athletes because of limited accessibility due to their strict and busy schedules. In this study, the availability of samples from elite athletes who belong to different sport disciplines was made possible via accessing samples from anti-doping laboratories. Furthermore, the screening of a wide variety of pro and anti-inflammatory cytokines and growth factors in conjunction with oxidative stress markers has enabled a comprehensive coverage of pathways associated with various exercise regimens, which resulted in the identification of the novel associations described by the emerging data.

## Conclusion

In this study, we have shown that certain markers of inflammation and oxidative stress vary between different sport groups. Compared to lower power sports, high power sports exhibit immune-inflammatory and oxidative stress markers associated with greater oxidative stress and less anti-inflammatory profile, potentially lowering their tissue healing capacity. Additionally, lower CD sports (footballers and athletics) were found to be associated with higher levels of pro-inflammatory cytokines compared to higher CD sports, perhaps reflecting enhanced muscle injury associated with the former sports. These differences require further validation to be utilized as potential biomarkers of athletes’ health, performance, and recovery from injury.

## Data Availability Statement

The datasets presented in this study can be found in online repositories. The names of the repository/repositories and accession number(s) can be found in the article/ Supplementary Material.

## Ethics Statement

The studies involving human participants were reviewed and approved by the Institutional Research Board of Qatar University. The patients/participants provided their written informed consent to participate in this study.

## Author Contributions

ME was responsible for the integrity of the work as a whole. All authors contributed to sample collection, analysis, manuscript writing and manuscript review and acceptance of final version.

## Conflict of Interest

The authors declare that the research was conducted in the absence of any commercial or financial relationships that could be construed as a potential conflict of interest.
